# Genetic conservation and management of the California endemic, Torrey pine (*Pinus torreyana* Parry): Implications of genetic rescue in a genetically depauperate species

**DOI:** 10.1002/ece3.3306

**Published:** 2017-08-09

**Authors:** Jill A. Hamilton, Raphaël Royauté, Jessica W. Wright, Paul Hodgskiss, F. Thomas Ledig

**Affiliations:** ^1^ Department of Biological Sciences North Dakota State University Fargo ND USA; ^2^ Pacific Southwest Research Station USDA‐Forest Service Davis CA USA; ^3^ Department of Plant Science University of California Davis CA USA

**Keywords:** common garden, genetic rescue, hybridization, *Pinus torreyana*, rare

## Abstract

Rare species present a challenge under changing environmental conditions as the genetic consequences of rarity may limit species ability to adapt to environmental change. To evaluate the evolutionary potential of a rare species, we assessed variation in traits important to plant fitness using multigenerational common garden experiments. Torrey pine, *Pinus torreyana* Parry, is one of the rarest pines in the world, restricted to one mainland and one island population. Morphological differentiation between island and mainland populations suggests adaptation to local environments may have contributed to trait variation. The distribution of phenotypic variances within the common garden suggests distinct population‐specific growth trajectories underlay genetic differences, with the island population exhibiting substantially reduced genetic variance for growth relative to the mainland population. Furthermore, F1 hybrids, representing a cross between mainland and island trees, exhibit increased height accumulation and fecundity relative to mainland and island parents. This may indicate genetic rescue via intraspecific hybridization could provide the necessary genetic variation to persist in environments modified as a result of climate change. Long‐term common garden experiments, such as these, provide invaluable resources to assess the distribution of genetic variance that may inform conservation strategies to preserve evolutionary potential of rare species, including genetic rescue.

## 1 INTRODUCTION

Species evolutionary potential is linked to the interplay between gene flow, mutation, genetic drift, and selection, impacting both the amount and distribution of genetic variation important to adaptation. In rapidly changing environments, the ability to evolve depends largely on the distribution of this variation, both within and across populations (Bridle & Vines, 2006; Hoffmann et al., 2015; Sgro, Lowe, & Hoffmann, 2011). Thus, major outstanding questions for rare species ask whether rare species have the necessary genetic variation to evolve and how that variation is distributed. Maintenance of evolutionary potential in rare species represents a challenge, particularly as these species are often isolated and exhibit reduced population size, exacerbating the genetic consequences of rarity. These consequences include an increased potential to exhibit reduced genetic variation due to the random loss of variation through drift, an increased probability of inbreeding, or reduced influx of variation via gene flow due to isolation (Ellstrand & Elam, 1993; Frankham, 2015; Hedrick, 2001). Reduced genetic variation can have direct consequences to long‐term population‐level fitness (Tallmon, Luikart, & Waples, 2004). If standing genetic variation and mutation alone provide limited adaptive capacity, rarity and isolation may contribute to increased risk of species’ extirpation (Frankham, 1998; Tallmon et al., 2004). In these scenarios, genetic or evolutionary rescue via managed introduction of genetic variation between populations may be required to conserve evolutionary potential of a species (Carlson, Cunningham, & Westley, 2014; Hamilton & Miller, 2016; Miller & Hamilton, 2016; Rius & Darling, 2014).

Genetic rescue, defined as the intentional immigration of new alleles between populations leading to increased population fitness and associated demographic vital rates, as a tool to limit biodiversity loss has received much attention to date, but limited application in the fields of conservation (Rius & Darling, 2014; Sgro, Lowe, & Hoffmann, 2010; Whitely, Fitzpatrick, Funk, & Tallmon, 2015). One of the reasons for this is that maintenance of species’ evolutionary potential via genetic rescue may have opposing effects in species of conservation concern (Kovach, Luikart, Lowe, Boyer, & Muhlfeld, 2016; Miller & Hamilton, 2016). On the one hand, the introduction of novel variation may limit demographic and genetic consequences of limited population size, providing the necessary variation to adapt to changing conditions (Carlson et al., 2014; Hufbauer et al., 2016). In their review of empirical studies of the effect of genetic rescue, Tallmon et al. (2004) suggested that even limited gene flow may contribute to increased fitness of small, inbred populations by increasing adaptive potential. However, outbreeding depression or the loss of unique genotypes following the introduction of novel variation remains a concern to conservation efforts, particularly where it leads to reduced fitness of recombinant individuals (Frankham et al., 2011; Hamilton & Miller, 2016; Rhymer & Simberloff, 1996). Even in cases where increased fitness attributable to heterosis is observed in an F1 generation, this advantage may not be maintained in later generations (Keller et al., 2001). Reduced fitness of F2s and beyond may result from intrinsic genetic incompatibilities leading to decreased fitness of recombinant genotypes (Johansen‐Morris & Latta, 2006; Schluter & Conte, 2009). Given the varied outcomes of genetic rescue, evaluating its consequences empirically in species of conservation concern is needed to develop long‐term species management programs that aim to restore or maintain evolutionary potential of species at risk.

Restricted to two discrete populations, Torrey pine (*Pinus torreyana* Parry), an iconic California endemic, is one of the rarest pine species in the world (Critchfield & Little, 1966). Torrey pine occupies a mainland grove of approximately 3,400 trees just north of San Diego in La Jolla, CA, at the Torrey Pines State Natural Reserve, as well as an island population of approximately 2,000 trees approximately 280 km on Santa Rosa Island, one of the Channel Islands (Franklin & Santos, 2011; Haller, 1986). Both populations are associated with coastal pockets of California, including fog and humidity characteristics critical to their survival (Ledig & Conkle, 1983). Previous genetic analyses based on allozyme genotyping (Ledig & Conkle, 1983) and chloroplast sequencing (Whittall et al., 2010) indicate that, at the studied loci, these trees are genetically identical, except for a few fixed differences found between the populations. This degree of monomorphism is virtually unknown in conifers and represents a substantial conservation concern. Within‐population genetic uniformity may leave trees increasingly vulnerable to pests, pathogens, and environmental change (Hoffmann & Sgro, 2011; Ledig & Conkle, 1983). Increased susceptibility to demographic stochasticity, loss of genetic diversity, and reduced adaptive potential is predicted to have dramatic consequences on plant performance, potentially leading to reduced fitness of Torrey pine populations under climate change scenarios (Anderson, 2016; Bijlsma & Loeschcke, 2012).

As limited genetic variation has been observed in Torrey pine using molecular markers, evaluating phenotypic variability across populations in a common environment may provide a measure of the genetic variation underlying traits important to plant performance. Heritable genetic variation in quantitative traits provides the raw material necessary for evolutionary responses, providing a quantifiable measure of evolutionary potential in a species (Davis, Shaw, & Etterson, 2005; Santiso et al., 2015). Thus, common garden experiments provide an important means to evaluate and partition genetic differences in adaptive traits within and among populations. In addition, evaluating the fitness consequences associated with interpopulation gene flow using F1 hybrids representing a cross between mainland and island trees provides an opportunity to directly assess the consequences of genetic rescue in this rare species. Evaluation of phenotypic differentiation across mainland, island, and F1 individuals will be crucial to predicting the impact intraspecific hybridization may have on evolutionary potential associated with long‐term survival of the species.

This study aims to evaluate the evolutionary potential of Torrey pine to inform conservation and restoration strategies for this rare species. Specifically, we test the prediction that in isolation, distinct evolutionary trajectories have contributed to population‐specific trait variation. We predict that morphological differentiation across populations may be associated with genetic differences important to adaptation within local environments. Given the degree of monomorphism described previously within Torrey pine, we predict isolation and rarity may lead to reduced genetic variance in traits important to plant fitness, limiting evolutionary potential under changing environmental conditions. Thus, to conserve evolutionary potential, we evaluate the impact genetic rescue may have, comparing fitness of F1 hybrids alongside parental populations. We predict increased genetic variance in hybrids for traits important to plant fitness may preserve the necessary variation to adapt to novel environments, enabling long‐term evolution in response to environmental change. Combined, this research will help inform ex situ and in situ conservation planning efforts and provide a means to begin to evaluate the potential of genetic rescue within this genetically depauperate long‐lived species.

## 2 MATERIALS AND METHODS

### 2.1 Plant material for multigenerational common garden experiments

Before 1960, the USDA Horticultural Field Station (now the Scripps Institute) at La Jolla, California, maintained Torrey pine trees originating from both mainland and island populations in two plots of 20 mainland and 20 island trees adjacent to each other (Haller, 1967). Seeds from Santa Rosa Island were collected by Guy Fleming, the first director of the Torrey Pines State Natural Reserve, and planted alongside a mainland collection of seeds. Little information remains regarding the details of the initial collection, experimental design, and establishment of this trial; however, a number of these trees persist and have produced cones, providing a second generation of progeny for further study. In 2004, open‐pollinated cones were collected from both island and mainland trees at the Scripps Institute to establish a second‐generation common garden experiment. The broad goal of the second‐generation experiment was to test for genetic and morphological differences that may have evolved between Torrey pine populations. Cones were first evaluated for differences associated with morphology. Measurements of island (two cones per nine maternal plants) and mainland trees (*n* = 10 bulk individuals) including height, diameter at breast height (DBH), cone weight, length and width of cone, umbo, and spine were taken based on recommendations from Haller (1986) and Johnson, Vander Wall, and Borchert (2003). Width‐to‐length ratio calculations were included to complement individual tree measurements. Needle morphology was examined for three needles per individual tree. Measurements included color and hue of needles, needle length, number of needles/fascicle (N/F), number of stomatal rows per curved face (SR/CF), stomatal rows per flat face (SR/FF), stomates per 10 ocular units curved face (S/10CF), stomates per 10 ocular units flat face (S/10FF), number of teeth per 10 ocular units (Teeth/10), and width of flat face (radius) per width of curved face (chord; Width R/C). Needle measurements were taken using a 4.9 stage micrometer at 2.5×, which is equivalent to 12 ocular units.

Seeds from this bulk collection of cones from 10 mainland trees (*n*_seeds_ = 128) and 16 individually identified open‐pollinated island trees (*n*_seeds_ = 515) were prepared for establishment of the second‐generation common garden experiment. Seeds were weighed and soaked in an aerated bath. Following this, seeds were planted in May 2006 in a shade‐controlled glasshouse at the USDA‐Forest Service Pacific Southwest Research Station, Institute of Forest Genetics in Placerville, CA, in D‐40 containers (10 in. height, 2.5 in. diameter, soil volume 40 in^3^) in a custom mix soil of 1 part clay loam to 2 parts sand with a seedling border row placed around the experimental trees. Seedlings were watered once per week, in general, with the watering regime erring on the dry side. Racks were rotated on a weekly basis to avoid potential effects of differential drying or shadowing of glasshouse benches. Ambient temperatures were meant to reflect average maximum (daytime) and minimum temperatures in San Diego, although outside air temperatures were influenced by the Placerville location, which is warmer in the summer months than either the mainland or island origin.

As seedlings emerged and shed their seed coat, one cotyledon was removed for genetic analysis using population‐specific allozyme markers. Previous work by Ledig and Conkle (1983) identified two allozyme loci fixed for alternate alleles between the mainland and island populations of Torrey pine with expected hybridization rates determined from a previous experiment (Table S1). Seedlings were classified as mainland, island, or F1 hybrid, representing an intraspecific hybrid between island maternal parents fertilized by mainland paternal pollen. Using the allozyme assay, 128 seedlings planted from the mainland bulk collection were genetically confirmed as mainland individuals, and the 515 seedlings, representing open‐pollinated island individuals, were genetically identified as pure island (*n* = 134) or F1 hybrid individuals (*n* = 381). Early life‐history characteristics of these seedlings were taken in the glasshouse comparing mainland, island, and F1 hybrid individuals prior to field planting. Data were recorded on germination date, number of cotyledons, and cotyledon length.

A subset of the island, mainland, and F1 hybrid seedlings were moved to Montecito, CA (34.4,538°N, 119.7,094°W), on 22 May 2007 and transplanted into a common garden. Seedlings were moved from the shade‐controlled glasshouse to an open‐air lath house to harden off and following transport were maintained in an open‐air shade house at the Santa Barbara Botanic Garden until planted at the Montecito site. Trees were planted on a 12 × 12 ft grid, with the same border row previously described surrounding the experiment and with competing brush removed prior to planting. Drip irrigation lines were run to each planting spot and watered prior to planting. A randomized complete block design was used for the common garden with six replicate blocks. Each block included a bulk collection of 20 mainland seedlings and 10 island maternal trees representing either two genetically confirmed pure island or two F1 hybrid seedlings per island mother for a total of 20 island and 20 hybrid seedlings of known maternal origin. In total, 360 seedlings were transplanted and examined for quantitative traits, including height and fecundity as measured by conelets produced, within the field common garden annually between 2008 and 2016.

### 2.2 Statistical analyses

To summarize the difference between mainland and island populations, variation in needle and cone morphological traits within the first‐generation common garden was summarized using pairwise *t* tests and a principal components analysis in R (R Development Core Team 2015).

Differences in seed weight, the number of days to germination, and cotyledon length were compared between island, mainland, and F1 hybrid individuals in the second‐generation progeny planting using ANOVA. In addition, these early life‐history traits were correlated with later‐life growth characteristics using Pearson correlations. These correlations were used to evaluate the relationship between seedling development across life‐history stages.

Variation in annual height accumulation was assessed using a repeated‐measures analysis that included an effect of year. The goal of this analysis was to partition the phenotypic variance within and among mainland, island, and hybrid individuals. A linear mixed‐effect model within the package *lme4* was used to test for population × year interactions with tree height (Bates, Maechler, Bolker, & Walker, 2014). We included population, year, and their interaction as fixed effects. Year was centered around its mean value. Individual trees, maternal identity (where possible), and block were included as random effects to control for additional sources of variation. We determined statistical significance of the fixed effects based on likelihood ratio tests and by computing the 95% confidence intervals (CI) using the likelihood profile.

A Bayesian hierarchical mixed model with the package MCMCglmm (Hadfield, 2010) was used to compare whether mainland, island, or hybrid populations differed in height variance. Because height was measured repeatedly over multiple years, we partitioned the phenotypic variation in height into *among‐ and within‐individual sources of variation: V*_P_ = *V*_ID_ + *V*_WI_, where *V*_P_ represents the total phenotypic variance, *V*_ID_ the among‐individual variance, and *V*_WI_ the within‐individual variance. Among‐individual variation arises through the combined influence of both additive genetic and permanent environment influences, while within‐individual variation is related to short‐term environmental variation (Boake, 1989; Dingemanse & Dochtermann, 2014). In the case of a common garden experiment, differences in among‐individual variance can serve as a proxy for differences in additive genetic variance because the permanent environment is shared by each population in the randomized planting design. This allows us to evaluate variation in height differences between populations and detects whether shifts in phenotypic variance are primarily a result of genetic or environmental influences.

As above, population, year, and their interactions were included as fixed effects. Individual trees were included as a random effect, and variance components were estimated separately by population to permit comparison of trait variances. Because both maternal identity and block variance were negligible in our analyses on population growth rate differences, these effects were not included in the present model. We specified an MCMC chain with 1.3 × 10^6^ iterations, 300,000 burn‐in period, and a thinning of 1,000 with use of a flat uninformative prior for the variance. We calculated the repeatability (*R*) of height variation as the proportion of the phenotypic variance resulting from among‐individual differences: *R* = *V*_ID_/*V*_P_, where *V*_P_ = *V*_ID_ +* V*_WI_ (the phenotypic, among‐, and within‐individual variances, respectively). We reported our estimates as the posterior mode and 95% credible intervals (CRI) of each component (Nakagawa & Schielzeth, 2010). We also calculated the MCMC posterior distribution for the difference in phenotypic variance between the island and hybrid populations relative to the mainland population (∆*V*_P Island_ = *V*_P Island_ − *V*_P Mainland_ and ∆*V*_P Mainland_ = *V*_P Hybrid_ − *V*_P Mainland_). This metric provides an estimation of the effect size for the change in phenotypic variance, with negative values indicating lower phenotypic variance compared to the mainland population and positive values greater phenotypic variance relative to the mainland population (Royaute, Buddle, & Vincent, 2015). “Significance” and inference of this difference were based on the overlap of credible intervals with zero and the proportion of estimates excluding zero (P). We repeated this procedure on each variance component and repeatability (∆*V*_ID_, ∆*V*_WI_, and ∆*R*) to determine whether changes in phenotypic variance were linked to changes in a specific variance component.

We also tested whether the correlation between tree fecundity, as measured by number of conelets produced, and height varied across populations. This permits a direct test of the assumption that height is a proxy for fitness in forest trees. The island population did not become reproductive during the course of our experiment; therefore, we specified a bivariate mixed model separately for just the mainland and hybrid population. Fecundity and height were used as response variables and individual trees as random effects. All fixed effects and model conditions were as above. This allowed us to estimate and compare among (*r*_ID_)‐ and within‐individual (*r*_WI_) correlation matrices between populations following Dingemanse and Dochtermann (2013). As above, we compared the magnitude of the difference in posterior estimates of correlation coefficients between populations (∆*r* = *r*_hybrid_ − *r*_mainland_) and based our inferences on the overlap of credible intervals with zero and the proportion of estimates excluding zero (P).

## 3 RESULTS

### 3.1 Adaptive trait differences among Torrey pine populations

**Figure 1:**
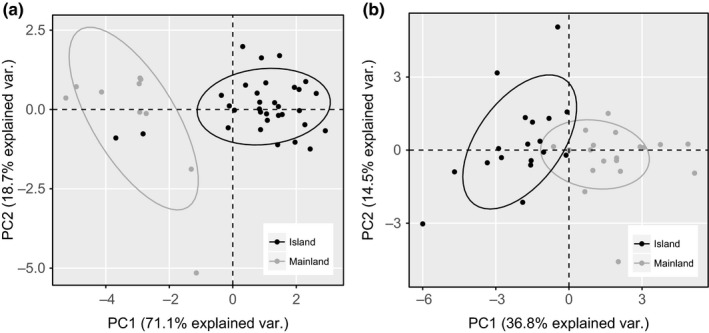
Principal components analysis for cone (a) and needle (b) morphological traits measured from mature island (black) and mainland (gray) Torrey pine trees planted together at the Scripps Institute

Needle and cone morphological traits measured in the first‐generation common garden were used to assess whether population‐specific morphological differences had evolved between mainland and island Torrey pine. The majority of morphological variation in cone and needle characteristics was explained by between‐population variation (Table S2; Figure 1). All cone morphological traits had positive loadings on the first principal component explaining 71.1% of the variation along the first axis, primarily distinguishing mainland from island populations (Figure 1a). Indeed, for all cone traits the island exhibited significantly increased size relative to the mainland population except for length (Table S3).

While there is more overlap between populations for needle morphological traits, PC1 explained 36.8% of the morphological variance, with the primary axis consistently differentiating the mainland from the island population (Figure 1b). Of the needle traits, only color and number of teeth per needle (Teeth/10) loaded negatively on PC1 and were not significantly different between populations (Table S3), indicating that mainland trees generally exhibit increased needle size and a greater number of stomata and associated traits, relative to island trees.

Early life‐history characteristics were compared among mainland, island, and F1 hybrid individuals within the second‐generation common garden experiment to assess developmental differences that may have evolved among Torrey pine populations. An analysis of variance indicated traits did differ significantly across populations based on significant pairwise differences identified from post hoc Tukey comparisons (Table 1; Fig. S1). Mainland seedlings germinated significantly earlier and had reduced seed weight relative to island and hybrid individuals. Cotyledon length was significantly greater in the hybrids relative to mainland and island individuals.

**Table 1:** ANOVA summarizing the distribution of variation for early life‐history growth between Torrey pine seedlings from mainland (M), island (I), or hybrid (F1) populations planted in a common glasshouse environment

Trait	Island	Hybrid (F1)	Mainland	*F*‐statistic	*p*‐value	Significant pairwise comparisons (population significantly different)
Number of days to germination	22.96 ± 4.4	23.58 ± 4.5	20.45 ± 3.1	26.14	<.001	M–H, M–I (Mainland)
Seed weight (g)	1.13 ± 0.2	1.16 ± 0.2	0.92 ± 0.2	90.37	<.001	M–H, M–I (Mainland)
Cotyledon length (mm)	65.2 ± 5.6	69.23 ± 5.5	65.58 ± 8.0	16.96	<.001	H–M, H–I (Hybrid)

Mean values for each trait provided along with standard deviation. Tukey's post hoc comparison identifies the significant pairwise comparison between populations (*p* < .05).

**Figure 2:**
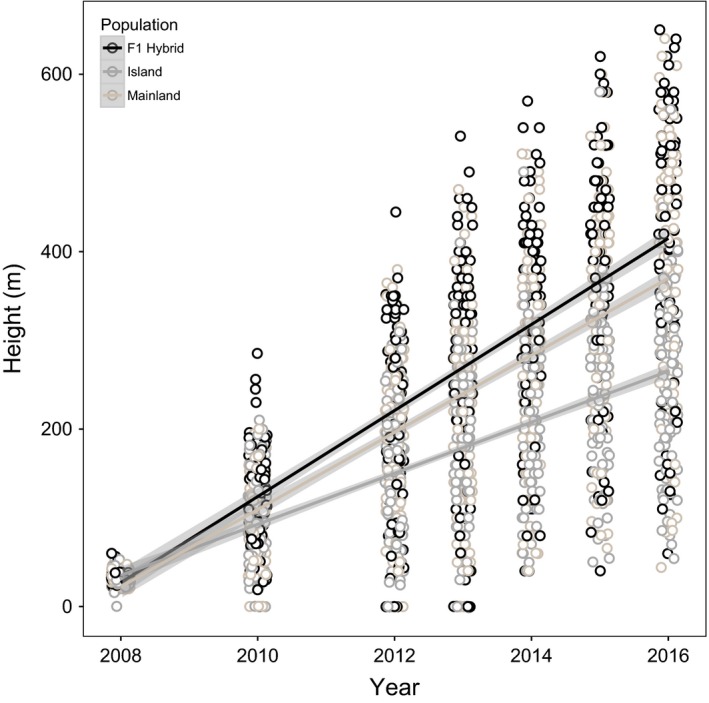
Growth of individual Torrey pine seedlings based on a repeated measure of height accumulated between 2008 and 2016 in the common garden experiment at Montecito. Height was evaluated independently for individuals within each population (island (light gray), mainland (medium gray), and F1 hybrid (black)) and used to estimate the growth rates (slope of each individual population)

Height accumulated per year varied substantially across mainland, island, and F1 hybrid trees within the second‐generation common garden (Figure 2). While all populations had accumulated largely similar height by 2008, we observed distinct trajectories as early as 2 years following field planting. On average, individuals with hybrid and pure mainland ancestry accumulated greater height per year than trees from the island population. Although the average height of F1 individuals was greater than mainland individuals, the rate of height accumulation did not differ significantly between these two populations. Island individuals, however, exhibited significantly lower height accumulation per year relative to the other two populations. Individuals of pure island ancestry exhibited a distinct growth trajectory relative to both mainland and hybrid progeny (Figure 2).

Pearson correlations between early life‐history characteristics measured in the glasshouse with later life‐history traits measured at the field common garden experiment, including height (2008) and fecundity (conelets 2013), suggest there is variability in the correlation of life‐history traits over time (Fig. S2). Seed weight, reflecting the maternal environment at the Scripps Institute, was not correlated with growth in the field. Correlations between seed weight with height (2008; *r *= 0.07) and conelet production (2013; *r *= −0.16) in the field common garden suggest that the maternal contribution via increased seed weight was not correlated with increased height accumulation and fecundity long‐term. Interestingly, time to germination and days to seed coat shed did not correlate with height in either the glasshouse or field experiment, indicating that traits associated with early phenological transitions may be independent of those traits characterizing growth and development. However, development following germination is likely correlated across time as height accumulation from the glasshouse (2006) was strongly correlated with height measurements in the field (2008; *r* = 0.44).

### 3.2 Differences in variance among populations

**Figure 3:**
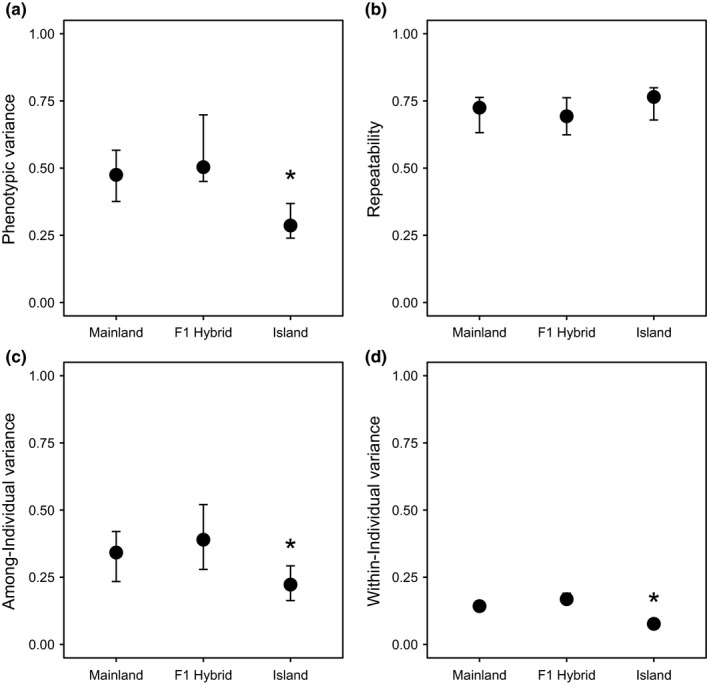
Phenotypic variance based on a Bayesian hierarchical mixed model of height repeatedly measured between 2008 and 2016 of mainland, island, and F1 hybrid Torrey pine populations. Variance is partitioned between the three populations into total phenotypic variance (a), repeatability of multiyear measurements (b), and variance attributable to among‐individual (c) and within‐individual sources of variation (d)

Variance partitioning for height was performed across the three populations teasing apart both among‐ and within‐individual variation. Phenotypic variance (*V*_P_) was significantly greater in hybrids relative to either the mainland or island population (Table S4; Figure 3a). Among‐individual variance was largely overlapping between the hybrid and mainland population; however, the island population exhibited significantly lower among‐individual variance (Figure 3b). The among‐individual variance arises from the genetic and permanent environment variance. While an index of relatedness is required to formally estimate additive genetic variances, as the permanent environmental variance is common to all three populations in the common garden, any change in the among‐individual variance across populations may be considered a proximate measure for additive genetic variance across populations (Boake, 1989; Dingemanse & Dochtermann, 2014). Reduced among‐individual variance (and thus proximately reduced additive genetic variance) relative to both the mainland and hybrid population suggests the island population may exhibit increased inbreeding or it could indicate that height is under strong selection in the island population.

In addition, within‐individual variance (*V*_WI_) was significantly lower within the island population (Figure 3). Reflecting the shorter‐term response to environmental variance, *V*_WI_ may provide some quantification of plasticity across populations in response to short‐term environmental heterogeneity. These results suggest that there is little within‐individual variance across all three populations, but the island had significantly lower within‐individual variance (Figure 3d). Low within‐individual variance should not be surprising, however, as height is expected to be a highly heritable trait (White, Adams, & Neale, 2007). Finally, overlapping repeatability of phenotypic measurements across years indicates the constancy of annual phenotypes measured across all populations over time (Figure 3b).

### 3.3 Impact of hybridization on trait means and variances

Variance components and their repeatability were evaluated for both the island and hybrid population relative to the mainland population (*V*_P Mainland_) to provide an effect size of hybridization. Negative effect sizes associated with the change in total phenotypic variance (−0.16 ∆*V*_P_) and within‐individual variance (−0.07 ∆*V*_WI_) indicates that there was a significant decrease in variance among island individuals relative to the mainland population (Table 2). Indeed, even the change in among‐individual variance (−0.11 ∆*V*_ID_) was significantly lower within the island relative to mainland (Table 2). In contrast, all effect sizes of hybrid individuals relative to the mainland were positive indicating greater variance in hybrids relative to mainland individuals. However, only the change in hybrid within‐individual variance was significantly greater than the mainland individuals (+0.02 ∆*V*_WI_), suggesting that the architecture of population‐level variance in height for mainland and hybrid populations may be largely similar.

**Table 2:** Changes in variance components and repeatability (*R*) between island (I–M) and hybrid (H–M) populations relative to the original mainland (M) population

	I–M		H–M	
	Estimate ± [95% CRI]	*p*	Estimate ± [95% CRI]	*p*
∆*V*_P_	**−0.17 **±** [−0.29; −0.06]**	.001	0.05 ± [−0.05; 0.25]	.12
∆*R*	0.04 ± [−0.05; 0.13]	.16	0.01 ± [−0.09; 0.09]	.48
∆*V*_ID_	*−0.08 ± [−0.22; 0.01]*	.04	0.04 ± [−0.08; 0.22]	.21
∆*V*_WI_	**−0.06 **±** [−0.09; −0.05]**	.00	**0.02 **±** [0.006; 0.06]**	.01

Bold indicates significance based on nonoverlap of the 95% credible intervals with zero, and italic indicates estimates for which the proportion of estimates excluding zero is below 0.05 (P).

As the island population had not yet become reproductively mature, correlations between height and fecundity were estimated for hybrid and mainland populations to test whether height is an appropriate fitness proxy in Torrey pine. Correlations between fecundity and height indicate a tight relationship among individuals for both mainland and hybrid populations with overlapping confidence intervals (Table 3). In contrast, the correlation decreases substantially for both mainland and hybrid within‐individual correlation matrices, with a positive effect of the change in correlation (∆*r* = 0.12) for hybrids relative to the mainland individuals. Increased within‐individual correlations between height and fecundity for hybrid populations implies that when admixed trees exhibit increased annual growth rates, they will also produce more conelets on that year.

**Table 3:** Correlation between tree fecundity as estimated by number of conelets and height providing the among (*r*_ID_)‐ and within‐individual (*r*_WI_) correlations matrices between populations

Population	*r*_ID_ ± [95% CI]	*r*_WI_ ± [95% CI]
Island	NA	NA
Mainland	0.69 [0.56; 0.81]	0.19 [0.06; 0.28]
Hybrid	0.73 [0.60; 0.84]	0.31 [0.19; 0.40]
∆*r* = *r*_hybrid_ − *r*_mainland_	0.04	0.12

The magnitude of difference in posterior estimates of correlation coefficients between populations is provided as ∆*r*.

## 4 DISCUSSION

The goal of this study was to ask broadly whether rare species of conservation concern have the necessary genetic variation to evolve in response to environmental heterogeneity. Restricted to two populations, the combination of isolation, small population size and lack of genetic variation suggests Torrey pine will be susceptible to human‐mediated and environmentally mediated stochasticity. Morphological differentiation between mainland and island populations indicates population‐specific traits may have evolved, a result of distinct evolutionary trajectories. Teasing apart the amount and distribution of genetic variance for a trait important to long‐term evolutionary potential suggests shifts in phenotypic variance are largely a result of genetic effects and erosion of genetic variation may have resulted, particularly significant within the island population. In contrast, F1 hybrids exhibited increased fitness relative to island and mainland populations. Thus, intraspecific genetic rescue may provide a mechanism to increase genetic variance and consequently demographic vital rates, preserving the long‐term evolutionary potential of the species. However, caution is still required. First, morphological differentiation may reflect adaptive differences that have evolved independently following isolation and genetic variation preserved within each population may be important to survival and reproduction in situ. Secondly, these results only reflect first‐generation F1 hybrids and heterosis may have contributed to F1 hybrid fitness. Genetic incompatibilities may cause hybrid breakdown in an F2 generation (Bomblies & Weigel, 2007; Hamilton, Lexer, & Aitken, 2013). Consequently, it will be important to assess the fitness of admixed individuals spanning multiple generations in their native environments to tease apart the longer‐term impacts genetic rescue may have.

### 4.1 Differentiation in morphological traits linked to adaptation to distinct environments

Torrey pine exhibits clear morphological differentiation associated with provenance of origin (Figure 1). Evaluation of cone and needle traits confirm previous observations of Haller (1986) that the two populations may be completely distinguished from each other based on morphological characteristics. This degree of differentiation is characteristic of pines with similarly geographically disjunct population distributions, including *Pinus muricata* and *P. radiata* (Haller, 1986), and likely has a genetic basis. However, whether these differences result from colonization history or selection following establishment or a combination of the two remains unclear (Haller, 1967).

The island population of Torrey pine produced substantially larger cones relative to their mainland congeners. Cone size may reflect adaptation to drier environments (Axelrod, 1982; Plessas & Strauss, 1986). Axelrod (1982) suggested that the evolution of large cone size was favored within isolated, increasingly xeric populations of Monterey pine (*Pinus radiata*) as a consequence of adaptation to drier conditions. Santa Rosa Island on average receives slightly more annual precipitation (331 mm) than the Torrey Pines State Natural Reserve (245 mm); however, evapotranspiration levels on the island (1,042 mm) are greater than those of the mainland population (1,024 mm), based on results from ClimateWNA (Wang, Hamann, Spittlehouse, & Murdock, 2012). Empirical examination of tree rings from different tree species across the Channel Islands suggests that growth is tightly linked to evapotranspiration rates and developmental strategies, such as seasonal photosynthetic activity, may have evolved to persist under limited water availability (Fischer & Still, 2007; Williams, 2006). Consequently, increased cone size may provide greater control of evapotranspiration rates within the island population, selecting for increased water use efficiency.

Adaptation to drier conditions has been implicated in a number of phenotypic differences in conifers, including reduced growth rates (Espinoza et al., 2014; Restaino, Peterson, & Littell, 2016; Teskey, Bongarten, Cregg, Dougherty, & Hennessey, 1987), prostrate growth habit (Haller, 1986), and physiological adaptations to control gas exchange, such as variation in stomatal densities and conductivity (Irvine, Perks, Magnani, & Grace, 1998; Sun, Livingston, Guy, & Ethier, 1996). Needle traits have evolved in a way that is consistent with adaptation under drier conditions. Regulation of water loss may be influenced by the density of stomata, particularly as plants exposed consistently to drought in sunlit conditions have evolved lower densities of stomata to reduce potential water loss (Grill, Tausz, Pollinger, Jimenez, & Morales, 2004). Reduced density of stomata among the island trees suggests that density of stomata may have been reduced in response to increased evapotranspiration. However, in contrast to cone morphology, where the majority of cone variation was explained by provenance (PC1 = 71.1%), provenance‐specific needle differentiation accounted for less variation (PC1 = 36.8%) indicating there is likely some plasticity associated with needle characteristics.

### 4.2 Early‐life development independent of established growth trajectory

The glasshouse/field common garden experiment reflects a second‐generation common garden experiment following a first‐generation parental common garden experiment. This experimental design provides the unique opportunity to control for the influence of maternal environment in a long‐lived tree. Given this design, the influence of maternal environment is likely extremely limited, reflected in the lack of correlation between seed weight and growth in the common garden (Fig. S1). In addition, evaluation of early life‐history traits alongside established growth trajectories provided a unique opportunity to evaluate shifts in developmental program following establishment. Interestingly, the mainland population exhibited significantly faster timing to germination and reduced seed weight relative to island and F1 hybrids (Fig. S1). Mainland seedlings germinated in advance of island seedlings and growth rates following germination were substantially lower in island seedlings. The island population may be adapted to limited resources, bounding its development (Larson, 1963). In addition, allocation to above‐ and belowground growth, not examined here, may contribute to differences observed. The harsh island environment may require trees to invest in belowground resources prior to aboveground to permit long‐term persistence (Jenkinson, 1977; Pinto, Marshall, Dumroese, Davis, & Cobos, 2016).

### 4.3 Growth of F1 hybrids reflects signature of heterosis in a novel environment

Repeated measures of height taken between 2008 and 2016 suggest island, mainland and hybrids have evolved genetic differences leading to distinct growth trajectories (Figure 2). F1 hybrids accumulated greater height per year than both the mainland and island population, with the island population exhibiting a significantly reduced rate of growth. The independent growth trajectory of the F1 hybrids likely reflect a signature of heterosis, increased fitness in response to mating between genetically divergent individuals (Rieseberg & Carney, 1998; Whitely et al., 2015). Interpopulation gene flow may maximize heterozygosity in the first‐generation (F1) hybrid, reducing excess homozygosity, thus masking possible signatures of inbreeding present within parental populations (Pickup, Field, Rowell, & Young, 2013; Williams & Savolainen, 1996). Inbreeding may be more prevalent in the island population as inbreeding erodes genetic variance. Thus, augmentation via genetic rescue may provide the necessary variation to reduce the fitness consequences of inbreeding depression within the island population, leading to increased growth rate and correlated fecundity (Keller & Waller, 2002; Richards, 2000).

Population origin may also have contributed to growth trajectories. Development of island individuals may reflect adaptation to the harsh island environment, particularly if resources are apportioned to promote persistence in drier environments (Williams, 2006; Williams, Still, Fischer, & Leavitt, 2008). Trees typically exhibit adaptive strategies that optimize resources and maximize competitive growth under different environmental conditions (Chmura et al., 2011; Wang, O'Neill, & Aitken, 2010). Strong selection following colonization may have led to a trade‐off between growth and fecundity for traits associated with water use efficiency, limiting growth potential within the island environment. Parallel growth trajectories observed between the mainland and F1 hybrids within the common garden may reflect environmental similarities between the mainland population and the novel environment of the common garden. The site at Montecito experiences slightly more annual precipitation based on ClimateWNA (381 mm). Increased precipitation may provide a competitive advantage for the mainland population as it exhibits an increased growth rate. However, as evapotranspiration rates are a better predictor of growth and coastal fog drip is likely the largest contributor of precipitation, evapotranspiration rates at the Montecito site (1,113 mm) may provide further selection for reduced growth in response to limited water availability akin to that observed on the island population (Fischer, Still, & Williams, 2009).

### 4.4 Change in the amount and distribution of phenotypic variance important to evolutionary potential

Evaluation of the amount and distribution of among‐individual variation for height indicates the island population has significantly lower among‐individual variation than the mainland population. In the case of this common garden experiment, the among‐individual variance serves as a proxy for additive genetic variance, reflecting a populations’ evolutionary potential. This suggests the island population may exhibit reduced evolutionary potential. Indeed, negative shifts in variance of the island relative to the mainland population for all variance components suggests the island may have reduced adaptive potential at least for height growth (Table 2). If reduced variation observed in height were the result of inbreeding, then we would except to see a similar reduction in variation across all traits. As island endemics traditionally have increased risk of extinction these results suggest the island population may be at greater risk of stochastic processes, including demographic, environmental, and catastrophic variation (Frankham, 1997; Santiso et al., 2015).

The observed reduced among‐individual genetic variance for height within the island population likely reflects a combination of the initial subset of the variation that persisted following founding and the combination of natural selection, random loss of variation due to drift, and increased levels of inbreeding (Frankham, 1998). Interestingly, among‐individual genetic effects were largely overlapping between the mainland and F1 hybrid population. In terms of conserving the genetic diversity of Torrey pine, based on this study, our results suggest the mainland population has a larger source of genetic variance and is thus likely to contribute disproportionately to the maintenance of evolutionary potential within the species as a whole. However, human sampling may have contributed to reduced genetic variance in the common garden, particularly if few maternal genotypes were sourced for mainland and island trees in the first‐generation parental garden at the Scripps site. Open‐pollinated seed sourced for the common garden at Montecito likely had a high potential for outcrossing for mainland and F1 individuals. Reduced genetic variance within island progeny may also be an artifact of few island genotypes planted, limiting the available gene pool. To address this possibility, we compared variance in the cone morphological traits between individuals of island ancestry from the Scripps Institute with a 2016 collection of individuals from Santa Rosa Island (Appendix S1). Individual variances of commonly measured traits were greater for those at the Scripps Institute than Santa Rosa Island, suggesting that the initial collection and establishment of island individuals at the Scripps Institute likely did not have reduced genetic variance relative to island population, biasing our estimates of genetic variance in their progeny.

Within‐individual variance for height across time (*V*_WI_) was low across all three populations. Although given the high degree of heritability in height for forest tree populations this is not surprising (White et al., 2007). The change in within‐individual variance within the hybrid population relative to the mainland population indicates hybrids may have greater opportunity to respond to short‐term environmental heterogeneity. Phenotypic plasticity is crucial to short‐term persistence and can produce genetic changes within a population important to adaptation (Ghalambor, McKay, Carroll, & Reznick, 2007). Loss of plasticity may be associated with the colonization of new environments leading to the evolution of specialization (Price, Qvarnstrom, & Irwin, 2003). In the case of the island population, phenotypic plasticity may be constrained as a result of adaptation to the local environment. Phenotypic plasticity increases the chance that individuals will survive in response to spatially and temporally varying environments (Silander, 1985). Indeed, plasticity may buffer limited genetic variation to permit increased adaptation to changing environmental conditions. Thus, reduced plasticity in the island population may confer a short‐term fitness disadvantage alongside longer‐term declines associated with reduced genetic variance. However, pollen donors from the native mainland population may have increased within‐individual genetic variance in the mainland and hybrid progeny collected from the Scripps Institute. Thus, reduced within‐individual variance within the island population may result from a smaller pool of pollen donors. Further testing of genetic variability and its distribution using contemporary genomic approaches is required to evaluate this theory.

### 4.5 Increased annual growth correlated with fecundity

Fecundity was highly correlated with height, suggesting that height for forest trees is generally a good proxy for fitness. However, the change in within‐individual correlations for hybrid individuals was positive relative to the mainland population. The tight relationship between height and fecundity indicate that differences observed across genotypes are under strong genetic control (Almqvist, Jansson, & Sonesson, 2001). Selection for increased growth rates in the hybrid population will have corresponding effects on fecundity, contributing to increased fitness of admixed individuals. These results may have important implications for implementing a genetic rescue program if the goal of the program is to increase the fitness of the species. However, it will be important to keep in mind that the signatures of F1 heterosis and correlations between height and fecundity reflect growth and development in the novel environment at Montecito. Natural selection may play an important role influencing development within the island and mainland environment. The island population has exhibited limited reproductive activity in the field common garden as of 2016, relative to the mainland and hybrid population. Delayed growth and reproduction may provide a competitive advantage within the island environment where adaptive developmental strategies may be necessary to persist under water‐limiting conditions. Williams (2006) suggested that the island population exhibits dramatically reduced photosynthetic activity in the summer months, an adaptive strategy in response to limited water availability and upper limits to annual growth. Without using reciprocal transplants to evaluate growth and development within the mainland or island environment, however, we cannot say that adaptation to local environments has not played an important role influencing growth rates within the current common garden. Given the species is at the limit of its range it may be a strong candidate for assisted migration (Richardson et al., 2009; Schwartz et al., 2012). In this case, it may be that hybrid populations may be important to consider for replanting outside the native environment.

### 4.6 Conservation of evolutionary potential in a rare species

One of the major outstanding questions in the conservation of rare species is whether these species have the necessary variation to evolve in response to changing environmental conditions. This study indicates population variance can be highly structured. There is within‐population variability; however, the mainland population harbors the majority of variability and the island population exhibits significantly reduced variation. Consequently, conservation management strategies may consider maintenance of the locally adapted diversity within the mainland and island populations. Additional studies are needed to determine if a genetic rescue for the island population would be supported. Trait differentiation across populations suggests that populations may have evolved in isolation. Adaptation to native environments is an important consideration in implementing management strategies.

Within the novel environment of the common garden, F1 hybrids appear more fit, on average, than either mainland or island population, indicating a genetic rescue may have fitness benefits. However, whether fitness of the F1s results from heterosis or the novel environment provides a competitive advantage for F1s remains to be tested. Ex situ plantings of mainland and island individuals will both conserve native germplasm and provide a means to empirically test fitness of Torrey pine populations in a range of environments, particularly important as Torrey pine is a candidate for assisted migration. Furthermore, as the signature of heterosis is often short‐term, it will be important to evaluate whether fitness advantages observed in the F1 are maintained in the F2 generation, resulting in adaptive introgression (Hamilton & Miller, 2016; Rieseberg, Archer, & Wayne, 1999; Whitely et al., 2015). In addition to establishing ex situ plantings of pure parents, progeny with mixed ancestry produced from mainland and F1 individuals within this experiment provide ideal material to test the longer‐term consequences of genetic rescue. As F1s reflect unidirectional gene flow from mainland pollen to island maternal trees it will be important to identify whether barriers to reproduction have evolved between these populations. The applicability of genetic rescue may be hindered if either extrinsic or intrinsic barriers to reproduction have formed between populations. Evaluation of genomic variation among parental, F1, and F2 generations of Torrey pine will provide the opportunity to compare demographic and evolutionary history of populations, with the potential to identify regions of the genome that may have diverged following isolation.

## CONFLICT OF INTEREST

None declared.

## AUTHOR CONTRIBUTIONS

JAH contributed to field assessments, data analysis, writing and editing of the manuscript; RR contributed to data analysis, writing and editing of the manuscript; JWW contributed to field assessments, writing and editing of the manuscript; PH contributed to molecular analysis and editing of the manuscript; and FTL led conception, design, and establishment of the experiment as well as field assessments.

## DISCLAIMER

Any use of trade, product, or firm names is for descriptive purposes only and does not imply endorsement by the US Government.

## DATA ARCHIVING STATEMENT

Data for this study are available at: to be completed after manuscript is accepted for publication.

## References

[ece33306-bib-0001] Almqvist, C. , Jansson, G. , & Sonesson, J. (2001). Genotypic correlations between early cone‐set and height growth in Picea abies clonal traits. Forest Genetics, 8, 194–204.

[ece33306-bib-0002] Anderson, J. T. (2016). Plant fitness in a rapidly changing world. New Phytologist, 210, 81–87. 26445400 10.1111/nph.13693

[ece33306-bib-0003] Axelrod, D. I. (1982). Age and origin of the Monterey endemic area. Madrono, 29, 127–147.

[ece33306-bib-0004] Bates, D. M. , Maechler, M. , Bolker, B. , & Walker, S. (2014). lme4: Linear mixed‐effects model using Eigen and S4. R package version 1.1‐6.

[ece33306-bib-0005] Bijlsma, R. , & Loeschcke, V. (2012). Genetic erosion impedes adaptive responses to stressful environments. Evolutionary Applications, 5, 117–129. 25568035 10.1111/j.1752-4571.2011.00214.x PMC3353342

[ece33306-bib-0006] Boake, C. R. (1989). Repeatability: Its role in evolutionary studies of mating behavior. Evolutionary Ecology, 3(2), 173–182.

[ece33306-bib-0007] Bomblies, K. , & Weigel, D. (2007). Hybrid necrosis: Autoimmunity as a potential gene‐flow barrier in plant species. Nature Reviews Genetics, 8(5), 382–393. 10.1038/nrg2082 17404584

[ece33306-bib-0008] Bridle, J. R. , & Vines, T. H. (2006). Limits to evolution at range margins: When and why does adaptation fail? Trends in Ecology & Evolution, 22(3), 140–147. 17113679 10.1016/j.tree.2006.11.002

[ece33306-bib-0009] Carlson, S. M. , Cunningham, C. J. , & Westley, P. (2014). Evolutionary rescue in a changing world. Trends in Ecology & Evolution, 29(9), 521–530. 25038023 10.1016/j.tree.2014.06.005

[ece33306-bib-0010] Chmura, D. J. , Anderson, P. D. , Howe, G. T. , Harrington, C. A. , Halofsky, J. E. , Peterson, D. L. , … St. Clair, B. (2011). Forest responses to climate change in the northwestern United States: Ecophysiological foundations for adaptive management. Forest Ecology and Management, 261, 1121–1142.

[ece33306-bib-0011] Critchfield, W. B. , & Little, E. L. (1966). Geographic distribution of the pines of the world, edited by U. S. D. o. Agriculture. Washington, DC: Forest Service.

[ece33306-bib-0012] Davis, M. B. , Shaw, R. G. , & Etterson, J. R. (2005). Evolutionary responses to changing climate. Ecology, 86(7), 1704–1714.

[ece33306-bib-0013] Dingemanse, N. J. , & Dochtermann, N. A. (2013). Quantifying individual variation in behaviour: Mixed effect modelling approaches. Journal of Animal Ecology, 82, 39–54. 23171297 10.1111/1365-2656.12013

[ece33306-bib-0014] Dingemanse, N. J. , & Dochtermann, N. A. (2014). Individual behaviour: Behavioural ecology meets quantitative genetics In Charmantier A., Garant D., & Kruuk L. E. B. (Eds.), Quantitative genetics in the wild. Oxford: Oxford University Press.

[ece33306-bib-0015] Ellstrand, N. C. , & Elam, D. R. (1993). Population genetic consequences of small population size: Implications for plant conservation. Annual Review of Ecology and Systematics, 24, 217–242.

[ece33306-bib-0016] Espinoza, S. E. , Martinez, V. A. , Magni, C. R. , Ivkovic, M. , Santelices, R. E. , Guerra, F. P. , & Cabrera, A. M. (2014). Genetic control of growth biomass allocation, and survival under drought stress in Pinus radiata D. Don seedlings. Tree Genetics & Genomes, 10, 1045–1054.

[ece33306-bib-0017] Fischer, D. T. , & Still, C. J. (2007). Evaluating patterns of fog water deposition and isotopic composition on the California Channel Islands. Water Resources Research, 43, 1–13.20300476

[ece33306-bib-0018] Fischer, D. T. , Still, C. J. , & Williams, A. P. (2009). Significance of summer fog and overcast for drought stress and ecological functioning of coastal California endemic plant species. Journal of Biogeography, 36, 783–799.

[ece33306-bib-0019] Frankham, R. (1997). Do island populations have less genetic variation than mainland populations? Heredity, 78, 311–327. 9119706 10.1038/hdy.1997.46

[ece33306-bib-0020] Frankham, R. (1998). Inbreeding and extinction: Island populations. Conservation Biology, 12, 665–675.

[ece33306-bib-0021] Frankham, R. (2015). Genetic rescue of small inbred populations: Meta‐analysis reveals large and consistent benefits of gene flow. Molecular Ecology, 24(11), 2610–2618. 25740414 10.1111/mec.13139

[ece33306-bib-0022] Frankham, R. , Ballou, J. D. , Eldridge, M. D. B. , Lacy, R. C. , Ralls, K. , Dudash, M. R. , & Fester, C. B. (2011). Predicting the probability of outbreeding depression. Conservation Biology, 25, 465–475. 21486369 10.1111/j.1523-1739.2011.01662.x

[ece33306-bib-0023] Franklin, J. , & Santos, E. V. (2011). A spatially explicit census reveals population structure and recruitment patterns for a narrowly endemic pine, *Pinus torreyana* . Plant Ecology, 212, 293–306.

[ece33306-bib-0024] Ghalambor, C. K. , McKay, J. K. , Carroll, S. P. , & Reznick, D. N. (2007). Adaptive versus non‐adaptive phenotypic plasticity and the potential for contemporary adaptation in new environments. Functional Ecology, 21, 394–407.

[ece33306-bib-0025] Grill, D. , Tausz, M. , Pollinger, U. , Jimenez, M. , & Morales, D. (2004). Effects of drought on needle anatomy of *Pinus canariensis* . Flora, 199, 85–89.

[ece33306-bib-0026] Hadfield, J. D. (2010). MCMC methods for multi‐response generalized linear mixed models: The MCMCglmm R package. Journal of Statistical Software, 33, 1–22. 20808728 PMC2929880

[ece33306-bib-0027] Haller, J. R. (1967). A comparison of the mainland and island populations of Torrey pine. Paper read at Proceedings of the Symposium on the Biology of the California Islands, at Santa Barbara Botanic Garden, Santa Barbara.

[ece33306-bib-0028] Haller, J. R. (1986). Taxonomy and relationships of the mainland and island populations of *Pinus torreyana* (Pinaceae). Systematic Botany, 11, 39–50.

[ece33306-bib-0029] Hamilton, J. A. , Lexer, C. , & Aitken, S. N. (2013). Genomic and phenotypic architecture of a spruce hybrid zone (*Picea sitchensis* × *P. glauca*). Molecular Ecology, 22, 827–841. 22967172 10.1111/mec.12007

[ece33306-bib-0030] Hamilton, J. A. , & Miller, J. M. (2016). Adaptive introgression as a resource for management and genetic conservation under climate change. Conservation Biology, 30, 33–41. 26096581 10.1111/cobi.12574

[ece33306-bib-0031] Hedrick, P. W. (2001). Conservation genetics: Where are we now? Trends in Ecology & Evolution, 16(11), 629–636.

[ece33306-bib-0032] Hoffmann, A. A. , Griffin, P. , Dillon, S. , Catullo, R. , Rane, R. , Byrne, M. , … Sgro, C. M. (2015). A framework for incorporating evolutionary genomics into biodiversity conservation and management. Climate Change Responses, 2, 1–23.

[ece33306-bib-0033] Hoffmann, A. A. , & Sgro, C. M. (2011). Climate change and evolutionary adaptation. Nature, 470, 479–485. 21350480 10.1038/nature09670

[ece33306-bib-0034] Hufbauer, R. A. , Szucs, M. , Kasyon, E. , Youngberg, C. , Koontz, M. J. , Richards, C. , … Melbourne, B. A. (2016). Three types of rescue can avert extinction in a changing environment. Proceedings of the National Academy of Science USA, 112(33), 10557–10562. 10.1073/pnas.1504732112 PMC4547288 26240320

[ece33306-bib-0035] Irvine, J. , Perks, M. P. , Magnani, F. , & Grace, J. (1998). The response of *Pinus sylvestris* to drought: Stomatal control of transpiration and hydraulic conductance. Tree Physiology, 18, 393–402. 12651364 10.1093/treephys/18.6.393

[ece33306-bib-0036] Jenkinson, J. L. (1977). Edaphic interactions in first‐year growth of California Ponderosa pine, edited by U. F. S. Research. Berkeley, CA: Pacific Southwest Forest and Range Experiment Station.

[ece33306-bib-0037] Johansen‐Morris, A. D. , & Latta, R. G. (2006). Fitness consequences of hybridization between ecotypes of Avena barbata: Hybrid breakdown, hybrid vigor, and transgressive segregation. Evolution, 60(8), 1585–1595. 17017059

[ece33306-bib-0038] Johnson, M. , Vander Wall, S. B. , & Borchert, M. (2003). A comparative analysis of seed and cone characteristics and seed‐dispersal strategies of three pines in the subsection Sabinianae. Plant Ecology, 168, 69–84.

[ece33306-bib-0039] Keller, L. F. , Jeffery, K. J. , Arcese, P. , Beaumont, M. A. , Hochachka, W. M. , Smith, J. N. M. , & Bruford, M. W. (2001). Immigration and the ephemerality of a natural population bottleneck: Evidence from molecular markers. Proceedings of the Royal Society B: Biological Sciences, 268, 1387–1394. 11429139 10.1098/rspb.2001.1607 PMC1088753

[ece33306-bib-0040] Keller, L. F. , & Waller, D. M. (2002). Inbreeding effects in wild populations. Trends in Ecology & Evolution, 17, 230–241.

[ece33306-bib-0041] Kovach, R. P. , Luikart, G. , Lowe, W. H. , Boyer, M. C. , & Muhlfeld, C. (2016). Risk and efficacy of human‐enabled interspecific hybridization for climate‐change adaptation: Response to Hamilton and Miller 2015. Conservation Biology, 30, 428–430. 26918487 10.1111/cobi.12678

[ece33306-bib-0042] Larson, M. M. (1963). Initial root development of Ponderosa pine seedlings as related to germination date and size of seed. Forest Science, 9(4), 456–460.

[ece33306-bib-0043] Ledig, F. T. , & Conkle, M. T. (1983). Gene diversity and genetic structure in a narrow endemic, Torrey Pine (*Pinus torreyana* Parry ex Carr.). Evolution, 37(1), 79–85. 28568032 10.1111/j.1558-5646.1983.tb05515.x

[ece33306-bib-0044] Miller, J. M. , & Hamilton, J. A. (2016). Interspecies hybridization in the conservation toolbox: Response to Kovach et al. (2016). Conservation Biology, 30, 431–433. 26918380 10.1111/cobi.12677

[ece33306-bib-0045] Nakagawa, S. , & Schielzeth, H. (2010). Repeatability for Gaussian and non‐Gaussian data: A practical guide for biologists. Biological Reviews, 85, 935–956. 20569253 10.1111/j.1469-185X.2010.00141.x

[ece33306-bib-0046] Pickup, M. , Field, D. L. , Rowell, D. M. , & Young, A. (2013). Source population characteristics affect heterosis following genetic rescue of fragmented plant populations. Proceedings of the Royal Society B: Biological Sciences, 280, 20122058. 23173202 10.1098/rspb.2012.2058 PMC3574427

[ece33306-bib-0047] Pinto, J. R. , Marshall, J. D. , Dumroese, R. K. , Davis, A. S. , & Cobos, D. R. (2016). Seedling establishment and physiological responses to temporal and spatial soil moisture changes. New Forests, 47, 223–241.

[ece33306-bib-0048] Plessas, M. E. , & Strauss, S. H. (1986). Allozyme differentiation among populations, stands, and cohorts in Monterey pine. Canadian Journal for Forest Research, 16, 1155–1164.

[ece33306-bib-0049] Price, T. D. , Qvarnstrom, A. , & Irwin, D. E. (2003). The role of phenotypic plasticity in driving genetic evolution. Proceedings of the Royal Society B: Biological Sciences, 270, 1433–1440. 12965006 10.1098/rspb.2003.2372 PMC1691402

[ece33306-bib-0050] R Development Core Team . (2015). R: A language and environment for statistical computer. Vienna, Austria: URL https://www.R-project.org/.

[ece33306-bib-0051] Restaino, C. M. , Peterson, D. L. , & Littell, J. (2016). Increased water deficit decreases Douglas fir growth throughout western US forests. Proceedings of the National Academy of Science USA, 113(34), 9557–9562. 10.1073/pnas.1602384113 PMC5003285 27503880

[ece33306-bib-0052] Rhymer, J. M. , & Simberloff, D. (1996). Extinction by hybridization and introgression. Annual Review of Ecology and Systematics, 27, 83–109.

[ece33306-bib-0053] Richards, C. (2000). Inbreeding depression and genetic rescue in a plant metapopulation. The American Naturalist, 155, 383–394. 10.1086/303324 10718733

[ece33306-bib-0054] Richardson, D. M. , Hellmann, J. J. , McLachlan, J. S. , Sax, D. F. , Schwartz, M. W. , Gonzalez, P. , … Vellend, M. (2009). Multidimensional evaluation of managed relocation. Proceedings of the National Academy of Science USA, 106, 9721–9724. 10.1073/pnas.0902327106 PMC2694035 19509337

[ece33306-bib-0055] Rieseberg, L. H. , Archer, M. A. , & Wayne, R. K. (1999). Transgressive segregation, adaptation and speciation. Heredity, 83, 363–372. 10583537 10.1038/sj.hdy.6886170

[ece33306-bib-0056] Rieseberg, L. H. , & Carney, S. E. (1998). Plant hybridization. New Phytologist, 140, 599–624. 10.1046/j.1469-8137.1998.00315.x 33862960

[ece33306-bib-0057] Rius, M. , & Darling, J. A. (2014). How important is intraspecific genetic admixture to the success of colonising populations? Trends in Ecology & Evolution, 29, 233–242. 24636862 10.1016/j.tree.2014.02.003

[ece33306-bib-0058] Royaute, R. , Buddle, C. M. , & Vincent, C. (2015). Under the influence: Sublethal exposure to an insecticide affects personality expression in a jumping spider. Functional Ecology, 29, 962–970.

[ece33306-bib-0059] Santiso, X. , Lopez, L. , Gilbert, K. , Barreiro, R. , Whitlock, M. C. , & Retuerto, R. (2015). Patterns of genetic variation within and among populations in Arbutus unedo and its relation with selection and evolvability. Perspectives in Plant Ecology, Evolution and Systematics, 17, 185–192.

[ece33306-bib-0060] Schluter, D. , & Conte, G. L. (2009). Genetics and ecological speciation. Proceedings of the National Academy of Science USA, 106, 9955–9962. 10.1073/pnas.0901264106 PMC2702799 19528639

[ece33306-bib-0061] Schwartz, M. W. , Hellmann, J. , McLachlan, J. S. , Sax, D. F. , Borevitz, J. O. , Brennan, J. , … Zellmer, S. (2012). Managed relocation: Integrating the scientific, regulatory, and ethical challenges. BioScience, 62, 732–743.

[ece33306-bib-0062] Sgro, C. M. , Lowe, A. J. , & Hoffmann, A. A. (2010). Building evolutionary resilience for conserving biodiversity under climate change. Evolutionary Applications, 4, 326–337. 25567976 10.1111/j.1752-4571.2010.00157.x PMC3352557

[ece33306-bib-0063] Sgro, C. M. , Lowe, A. J. , & Hoffmann, A. A. (2011). Building evolutionary resilience for conserving biodiversity under climate change. Evolutionary Applications, 4, 326–337. 25567976 10.1111/j.1752-4571.2010.00157.x PMC3352557

[ece33306-bib-0064] Silander, J. A. (1985). The genetic basis of the ecological amplitude of Spartina patens. II. Variance and correlation analysis. Evolution, 39, 1034–1052. 28561514 10.1111/j.1558-5646.1985.tb00445.x

[ece33306-bib-0065] Sun, Z. J. , Livingston, N. J. , Guy, R. D. , & Ethier, G. J. (1996). Stable carbon isotopes as indicators of increased water use efficiency and productivity in white spruce (*Picea glauca* (Moench) Voss) seedlings. Plant, Cell and Environment, 19, 887–894.

[ece33306-bib-0066] Tallmon, D. A. , Luikart, G. , & Waples, R. S. (2004). The alluring simplicity and complex reality of genetic rescue. Trends in Ecology & Evolution, 19(9), 489–496. 16701312 10.1016/j.tree.2004.07.003

[ece33306-bib-0067] Teskey, R. O. , Bongarten, B. C. , Cregg, B. M. , Dougherty, P. M. , & Hennessey, T. C. (1987). Physiology and genetics of tree growth response to moisture and temperature stress: An examination of characteristics of loblolly pine (*Pinus taeda* L.). Tree Physiology, 3, 41–61. 14975834 10.1093/treephys/3.1.41

[ece33306-bib-0068] Wang, T. , Hamann, A. , Spittlehouse, D. , & Murdock, T. Q. (2012). ClimateWNA ‐ high‐resolution spatial climate data for western North America. Journal of Applied Meteorology and Climatology, 51, 16–29.

[ece33306-bib-0069] Wang, T. , O'Neill, G. A. , & Aitken, S. (2010). Integrating environmental and genetic effects to predict responses of tree populations to climate. Ecological Applications, 20(1), 153–163. 20349837 10.1890/08-2257.1

[ece33306-bib-0070] White, T. L. , Adams, W. T. , & Neale, D. B. (2007). Forest genetics. Cambridge: CABI Publishing.

[ece33306-bib-0071] Whitely, A. R. , Fitzpatrick, S. W. , Funk, W. C. , & Tallmon, D. A. (2015). Genetic rescue to the rescue. Trends in Ecology & Evolution, 30, 42–49. 25435267 10.1016/j.tree.2014.10.009

[ece33306-bib-0072] Whittall, J. B. , Syring, J. , Parks, M. , Buenrostro, J. , Dick, C. , Liston, A. , & Cronn, R. (2010). Finding a (pine) needle in a haystack: Chloroplast genome sequence divergence in rare and widespread pines. Molecular Ecology, 19, 100–114. 20331774 10.1111/j.1365-294X.2009.04474.x

[ece33306-bib-0073] Williams, A. P. (2006). Teasing foggy memories out of pines on the California Channel Islands using tree‐ring width and stable isotope approaches. Geography, University of California ‐ Santa Barbara, Santa Barbara.

[ece33306-bib-0074] Williams, C. G. , & Savolainen, O. (1996). Inbreeding depression in conifers: Implications for breeding strategy. Forest Science, 42, 102–117.

[ece33306-bib-0075] Williams, A. P. , Still, C. J. , Fischer, D. T. , & Leavitt, S. W. (2008). The influence of summertime fog and overcast clouds on the growth of a coastal Californian pine: A tree‐ring study. Oecologia, 156, 601–611. 18368424 10.1007/s00442-008-1025-y

