# Supplementary figures and images for: Genetic conservation and management of the California endemic, Torrey pine (Pinus torreyana Parry): Implications of genetic rescue in a genetically depauperate species
Source: Ecol Evol. 2017 Aug 9;7(18):7370–81. doi: 10.1002/ece3.3306 (PMC5606898; doi:10.1002/ece3.3306)

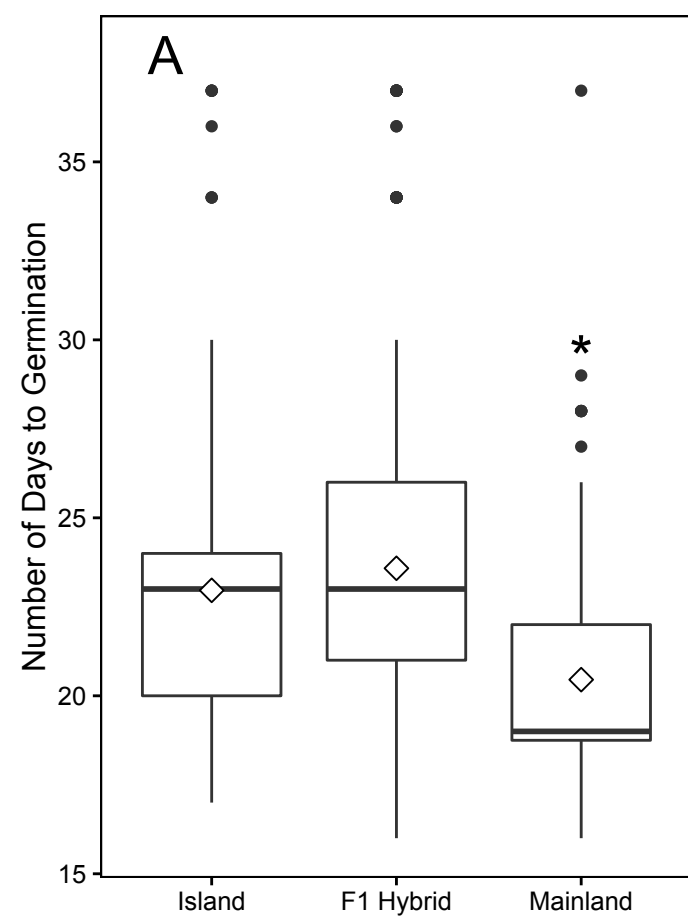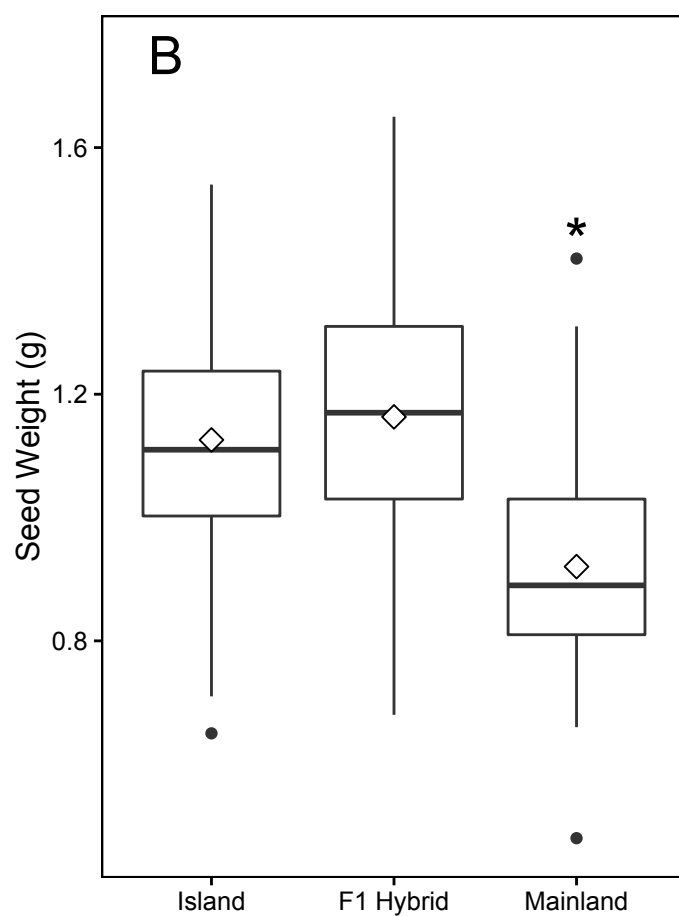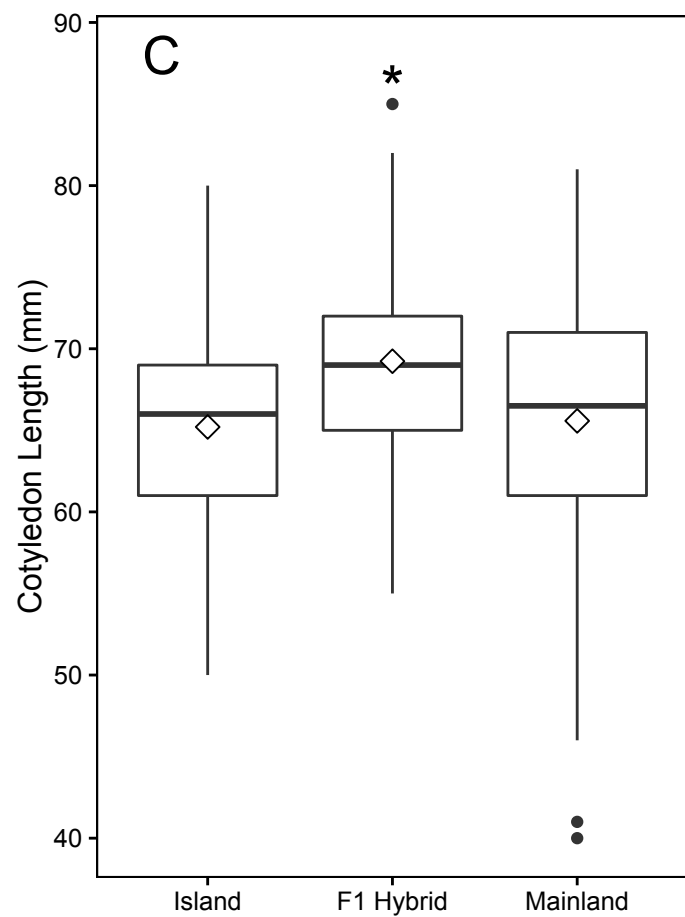

Supplement: Supplementary file 1 [file ECE3-7-7370-s001.pdf]

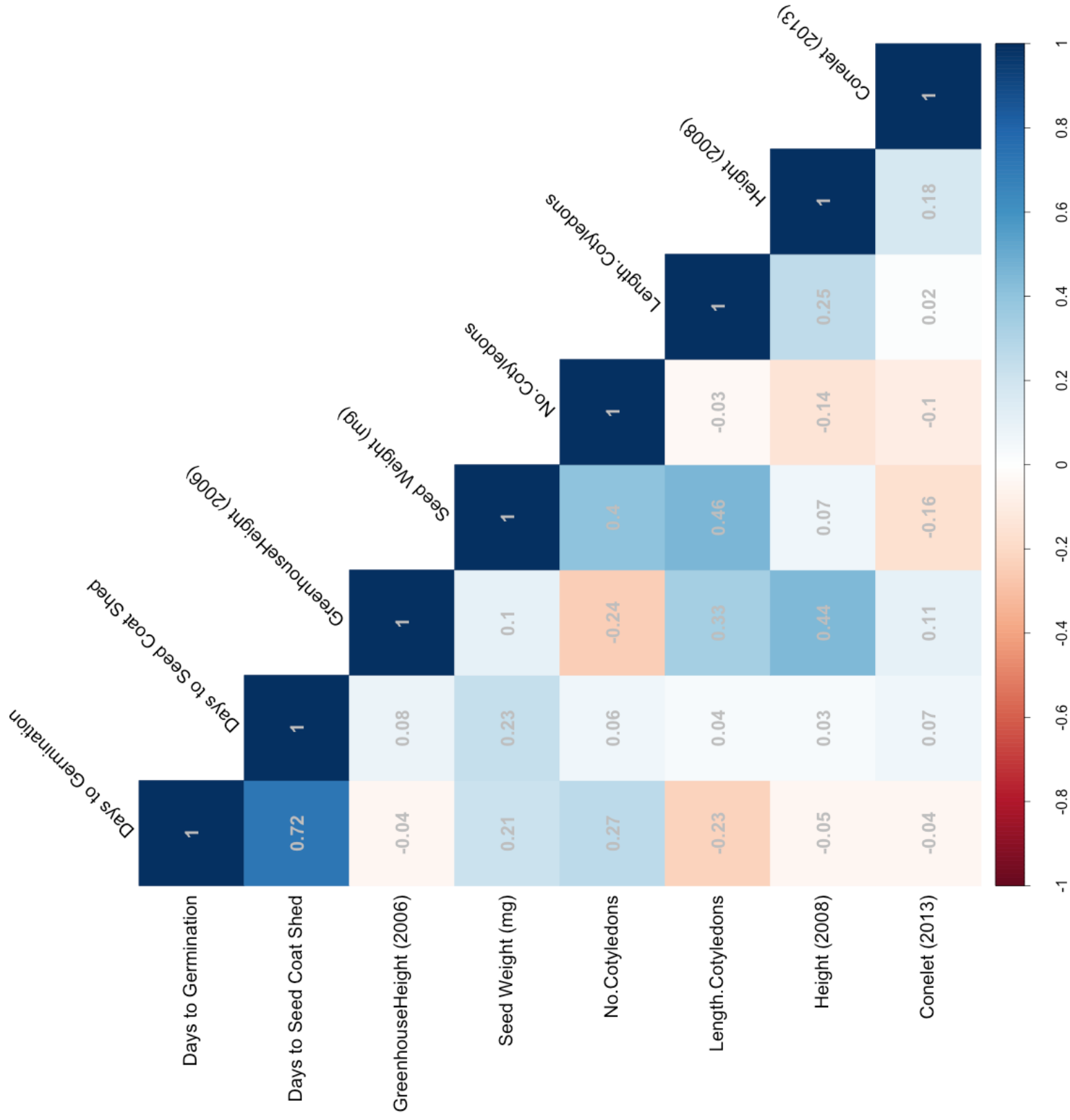

Supplement: Supplementary file 2 [file ECE3-7-7370-s002.pdf]
