# Supplementary material for: Genetic conservation and management of the California endemic, Torrey pine (Pinus torreyana Parry): Implications of genetic rescue in a genetically depauperate species
Source: Ecol Evol. 2017 Aug 9;7(18):7370–81. doi: 10.1002/ece3.3306 (PMC5606898; doi:10.1002/ece3.3306)
Supplement: Supplementary file 7 [file ECE3-7-7370-s007.docx]

**Supplementary Appendix for:** Genetic conservation and management of the California endemic, Torrey pine (*Pinus torreyana* Parry)

**Comparison of cone morphology between trees from Santa Rosa Island and the Scripps Institute common garden**

**Methods:**

We compared cone morphology variance between island individuals from the Santa Rosa Island population and the first-generation common garden experiment at the Scripps Institute as a means to determine whether the amount of genetic variation was comparable between the natural population and the common garden. Santa Rosa island individuals were sampled in November 2016 and included 20 trees with three cones sampled per tree and a total of 60 measurements. Cones from the Scripps institute were measured on 16 trees with two cones measured per tree for a total of 32 measurements. For each cone, we measured its total length and width (in mm) as well as the length and width of the umbo of two separate spines. We first performed a PCA on all six cone morphological measurements and tested for differences in overall morphology between both populations using t-test on the first two principal components. We then compared the differences in among and within-individual variation between both populations using linear mixed models for length, width as well as the first two principal components for morphology data. These mixed models were specified using the maximum likelihood-based package *nlme* with population as a fixed effect, tree identity as random effects and by allowing the among and residual variance (i.e. the within-individual variance) to be estimated separately for each population. This analysis is based on a relatively small sample size, therefore we did not perform significant tests but are merely interested in whether the value of among-individual variance is comparable between both populations. The among-individual variance is generated by both permanent environment and additive genetic sources of variation, and because permanent sources of variation are reduced within the common garden system, we expect values of among-individual variance to be comparable between both population if genetic variance in the Scripps Institute trees is at least as large as that of the natural population on the Santa Rosa Island.

**Results interpretation:**

Cones from the island population at the Scripps institute had larger umbo length and width compared to our measurements taken at the Santa Rosa island from 2016 (Umbo length 1: t = -49.04, P =<0.001, Umbo length 2: t=-23.98, t<0.001). However, cones did not differ on average in length and width (Cone length: t =0.868 , P =0.388, Cone width: t=-0.301, P=0.765) (Table A1, Fig. A1). We found little evidence for lower among-individual variance in our samples from the Scripps Institute, suggesting that variance at the Scripps Institute likely represents 16 different pairs of parents, and that a diverse array of island genotypes were used to establish the first-generation common garden. In fact among individual variance was always larger for the Scripps Institute traits compared to the Santa Rosa island traits (V_ID_ = 0.29 – 0.77, ΔV_ID_ > 0.29) for all cone measurements (Table A2). Note however that the total phenotypic variance and within-individual variances were generally larger in the Santa Rosa Island population (V_P_ = 0.11 – 2.22, -1.29 <ΔV_P_ < 0.32; V_WI_ = 0.08 – 2.08, -1.92 < ΔV_WI_ < -0.01). This is consistent with the interpretation of reduced environmental variation in the common garden population compared to the natural population occurring on the Santa Rosa Island.

**Tables**

**Table A1:** Loadings and variance explained by each principal component for cone morphological traits.

| Trait | PC1 | PC2 | PC3 | PC4 | PC5 | PC6 |
| --- | --- | --- | --- | --- | --- | --- |
| Length | 0.00 | 0.71 | 0.71 | 0.00 | 0.00 | 0.00 |
| Width | 0.00 | 0.71 | -0.70 | 0.00 | 0.00 | 0.00 |
| Umbo length 1 | -0.50 | 0.00 | 0.00 | -0.39 | 0.34 | -0.69 |
| Umbo width 1 | -0.50 | 0.00 | 0.00 | -0.48 | -0.66 | 0.31 |
| Umbo length 2 | -0.50 | 0.00 | 0.00 | 0.00 | 0.60 | 0.61 |
| Umbo width 2 | -0.49 | 0.00 | 0.00 | 0.785 | -0.3 | -0.227 |
| Variance | 1.96 | 1.29 | 0.58 | 0.33 | 0.24 | 0.07 |
| Variance explained | 0.64 | 0.28 | 0.06 | 0.02 | 0.01 | 0.00 |
| Cumulative variance | 0.64 | 0.91 | 0.97 | 0.99 | 1.00 | 1.00 |

**Table A2:** Variance partitioning for cone morphology compared between the Santa Rosa Island population and the population established as a common garden experiment at the Scripps Institute. Variance components were estimated for total phenotypic variance (V_P_), repeatability of annual phenotypic measure (R), among-individual variance (V_ID_), and within-individual variance (V_WI_) and their respective differences (Δ). Positive values of Δ indicate greater amount of variation in the population from the Scripps Institute common garden compared to the Santa-Rosa population.

|  | Scripps | | | |  | Santa-Rosa Island | | | |  | Δ  (Scripps-Santa-Rosa) | | | |
| --- | --- | --- | --- | --- | --- | --- | --- | --- | --- | --- | --- | --- | --- | --- |
| Trait | V_ID_ | V_WI_ | V_P_ | R |  | V_ID_ | V_WI_ | V_P_ | R |  | V_ID_ | V_WI_ | V_P_ | R |
| Length | 0.29 | 0.11 | 0.40 | 0.73 |  | 0.00 | 1.42 | 1.42 | 0.00 |  | 0.29 | -1.31 | -1.02 | 0.73 |
| Width | 0.51 | 0.10 | 0.60 | 0.84 |  | 0.26 | 1.05 | 1.30 | 0.20 |  | 0.25 | -0.95 | -0.70 | 0.65 |
| PC1 | 0.36 | 0.07 | 0.43 | 0.84 |  | 0.04 | 0.08 | 0.11 | 0.32 |  | 0.32 | -0.01 | 0.32 | 0.52 |
| PC2 | 0.77 | 0.16 | 0.93 | 0.83 |  | 0.14 | 2.08 | 2.22 | 0.06 |  | 0.63 | -1.92 | -1.29 | 0.76 |

**Figure A1.** Principal component analysis for cone morphological traits measured from mature Santa Rosa Island trees (SRI, green) and island trees planted at the Scripps Institute (red).
